# Modulation of walking speed by changing optic flow in persons with stroke

**DOI:** 10.1186/1743-0003-4-22

**Published:** 2007-06-26

**Authors:** Anouk Lamontagne, Joyce Fung, Bradford J McFadyen, Jocelyn Faubert

**Affiliations:** 1 School of Physical and Occupational Therapy, McGill University and Jewish Rehabilitation Hospital Research Center (CRIR), Montreal, Canada; 2 Department of Rehabilitation, Laval University, and Quebec Rehabilitation Research Institute (CIRRIS), Quebec, Canada; 3 Vision and Perception Laboratory, School of Optometry, University of Montreal, Montreal, Canada

## Abstract

**Background:**

Walking speed, which is often reduced after stroke, can be influenced by the perception of optic flow (OF) speed. The present study aims to: 1) compare the modulation of walking speed in response to OF speed changes between persons with stroke and healthy controls and 2) investigate whether virtual environments (VE) manipulating OF speed can be used to promote volitional changes in walking speed post stroke.

**Methods:**

Twelve persons with stroke and 12 healthy individuals walked on a self-paced treadmill while viewing a virtual corridor in a helmet-mounted display. Two experiments were carried out on the same day. In experiment 1, the speed of an expanding OF was varied sinusoidally at 0.017 Hz (sine duration = 60 s), from 0 to 2 times the subject's comfortable walking speed, for a total duration of 5 minutes. In experiment 2, subjects were exposed to expanding OFs at discrete speeds that ranged from 0.25 to 2 times their comfortable speed. Each test trial was paired with a control trial performed at comfortable speed with matching OF. For each of the test trials, subjects were instructed to walk the distance within the same time as during the immediately preceding control trial. VEs were controlled by the CAREN-2 system (Motek). Instantaneous changes in gait speed (experiment 1) and the ratio of speed changes in the test trial over the control trial (experiment 2) were contrasted between the two groups of subjects.

**Results:**

When OF speed was changing continuously (experiment 1), an out-of-phase modulation was observed in the gait speed of healthy subjects, such that slower OFs induced faster walking speeds, and vice versa. Persons with stroke displayed weaker (p < 0.05, T-test) correlation coefficients between gait speed and OF speed, due to less pronounced changes and an altered phasing of gait speed modulation. When OF speed was manipulated discretely (experiment 2), a negative linear relationship was generally observed between the test-control ratio of gait speed and OF speed in healthy and stroke individuals. The slope of this relationship was similar between the stroke and healthy groups (p > 0.05, T-test).

**Conclusion:**

Stroke affects the modulation of gait speed in response to changes in the perception of movement through different OF speeds. Nevertheless, the preservation of even a modest modulation enabled the persons with stroke to increase walking speed when presented with slower OFs. Manipulation of OF speed using virtual reality technology could be implemented in a gait rehabilitation intervention to promote faster walking speeds after stroke.

## Background

Regulation of walking involves the integration of visual, proprioceptive and vestibular information. Optic flow (OF) is a typical pattern of visual motion generated at the eye as the person moves through the environment [1]. OF is a source of visual information that can be used to control heading direction [2-4] and speed [5-8] of walking, as it provides information about the direction and speed of self-motion with respect to the environment. Studies in healthy individuals have shown that changing OF speed modulates walking speed while walking on a self-paced treadmill [6-9]. During walking, visual (OF) and proprioceptive information about self-motion are normally congruent. When OF speed is manipulated in such way that it mismatches the proprioceptive information from the legs, walking velocity is adjusted to reduce the incongruity. The strategy adopted by healthy subjects is to reduce their walking speed with an increasing speed of OF, and to increase their speed at slower OFs [6,7]. This adaptation has been shown to be altered by neurodegenerative diseases such as Parkinson's disease [10], in which the heavy reliance on visual [11] and kinesthetic information would produce exaggerated gait speed modulation responses.

Slow speed, a feature that characterizes locomotion after stroke [12], can impact on functional ability such as crossing a street within the time allotted by the pedestrian light. It is well accepted that muscle weakness, particularly on the paretic lower limb, is one of the main factors explaining slow gait speed after stroke [13,14]. When motivated and instructed to walk at faster speeds, however, subjects with stroke have the capacity to at least double their walking speed [15], suggesting that factors other than muscle weakness may be contributing. It has also been shown that the discrimination of direction of global motion is altered by stroke, even when the primary cortical visual areas are preserved [16,17]. Whether similar deficits in discriminating OF speed exist in this population is yet to be determined. The present study is based on the premise that an altered perception or integration of OF speed information could contribute to the difficulty of subjects with stroke in regulating walking speed. A residual ability to utilize OF to control the speed of walking, however, would provide the basis for gait intervention with virtual reality that manipulates OF parameters to enhance walking ability after stroke.

The specific aims of this study were: first, to compare the non-volitional modulation of walking speed in response to an OF of variable speed between persons with stroke and healthy controls and second, to investigate whether a virtual reality-based paradigm that manipulates OF speed could be used to promote volitional changes in walking speed in persons with stroke. We hypothesized that persons with stroke, although presenting with an altered modulation of walking speed in response to OFs of changing speed, would still present sufficient modulation such that a paradigm based on OF speed manipulation could be used to promote faster walking speeds.

## Methods

### Subjects

Twelve subjects with a hemiparesis (9 males/3 females) due to a first-time stroke in the middle cerebral artery region and twelve healthy controls (8 males/4 females) participated in this study (Table 1). The location of the stroke was confirmed by computerized tomography or magnetic resonance imaging. Subjects with stroke presented with mobility problems, as they were ambulating at a walking speed slower than 1 m/s, with or without walking aid. They had no visual field defect, as assessed by the optometrist using the Goldman Test or equivalent measure. All but 2 were free of visuospatial neglect (subjects S3 and S4), as assessed by the Bell's test [18] or Star Cancellation Test [19]. As those 2 subjects did not behave differently from the others, their data were pooled together with the other subjects with stroke for analysis. Any subject with orthopaedic or another neurological condition that could interfere with locomotion was excluded. All subjects signed an informed consent document and the project was approved by the Montreal Center for Interdisciplinary Research in Rehabilitation (CRIR).

**Table 1:** Subject Characteristics

**Stroke (n = 12)**	**Age (yrs)**	**Gender (/M)**	**Height (cm)**	**Weight (Kg)**	**Speed (m/s)**	**Side (R/L)**	**Time Stroke (mo)**	**Location CVA**
S1	50	M	173	75	0.80	R	3.4	Sylvian L
S2	79	M	176	73	0.35 (Cane)	L	27.7	Subcortical R
S3	71	M	178	72	0.15 (Quad*)	R	14.0	Sylvian L
S4	66	M	152	68	0.18 (Quad)	L	19.0	Sylvian R
S5	73		155	48	0.3 (Cane)	R	2.9	Sylvian L
S6	79	M	152	60	0.60 (2 Canes)	R	2.1	Sylvian L
S7	64	M	172	90	0.15 (Quad)	R	1.5	Sylvian L
S8	51	M	185	96	0.66	L	51.6	Sylvian R
S9	73		154	90	0.34	R	1.0	Sylvian L
S10	68	M	162	50	0.25 (Cane)	L	3.2	Sylvian R
S11	80	M	172	70	0.12(Quad)	L	2.1	Sylvian R
S12	67		180	88	0.85	L	3	Sylvian R
**Mean**	**68.4**	**-**	**168**	**73**	**0.40**	**-**	**11**	**-**
**Range**	**50–80**	**-**	**152–180**	**48–96**	**0.12–0.85**		**1.0–51.6**	**-**

**Controls**								
**(n = 12)**								
**Mean**	**63.5**	**-**	**169**	**79**	**1.12**	**-**	**-**	**-**
**Range**	**50–74**	**-**	**152–185**	**63–104**	**0.81–1.67**	**-**	**-**	**-**

### Set-up and Procedures

**Figure 1:**
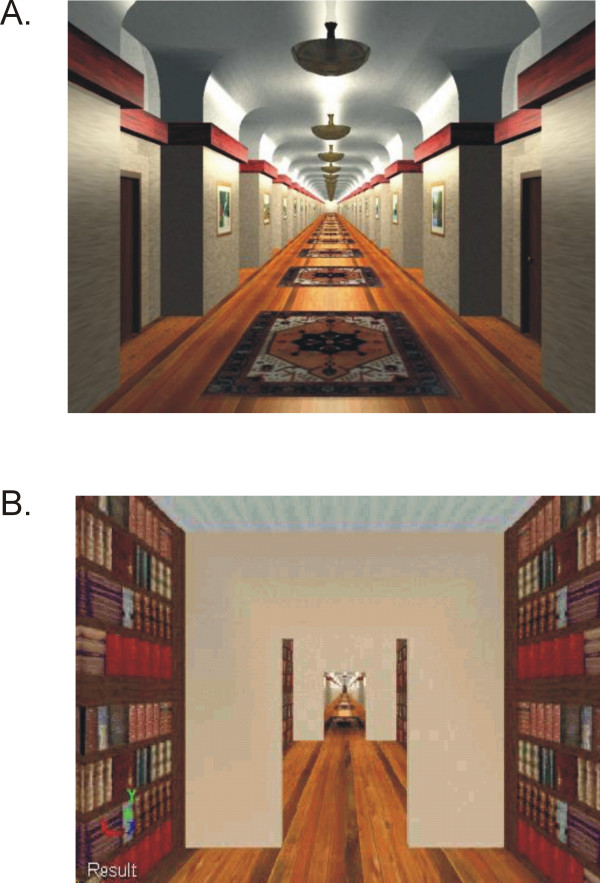
Virtual corridors used for Experiment 1 (A) and Experiment 2 (B). The scene represented in (A) is adapted from the library of VRCO Inc.

Subjects were evaluated while walking on a self-paced treadmill and watching, in a head mounted display (HMD), virtual environments representing corridors (Figure 1). The HMD (Kaiser Optics ProView™ XL50) has a field of view of 50° diagonal, 30° vertical by 40° horizontal. The self-paced treadmill allows one to modify walking speed at will, by servo-controlling the motor with a real-time algorithm that takes into account the pelvis position in the anteroposterior direction, as measured by a potentiometer tethered to the subject, and the walking speed. The self-paced treadmill was equipped with bilateral sliding handrails, which allowed arm swing and anteroposterior displacements of the body while assisting with balance maintenance during walking. A safety harness system suspended overhead prevented the subjects from falling.

Subjects were first habituated to walk on the self-paced treadmill without the HMD, and then with the HMD while watching a virtual corridor scene expanding at a speed matching their comfortable walking speed. Comfortable walking speed was determined after a minimum of 4 to 5 minutes of habituation while walking with the HMD. Subjects listened to white and pink noises through earphones during testing, to avoid any speed feedback from auditory cues. White noise has equal energy at all frequencies, while pink noise has more power in the lower frequencies that the higher frequencies. The latter provides additional masking power for low frequency noises.

Two series of experiments were conducted on the same day, with experiment 2 following experiment 1. Because of fatigue or time constraint, one healthy control and 3 subjects with stroke did not complete experiment 2. In the first experiment, the instantaneous and non-volitional changes in walking speed in response to an OF of continually changing speed (Experiment 1) were examined. In the second, the voluntary or intentional changes in walking speed in response to OFs at discrete constant speeds (Experiment 2) were examined. In *Experiment 1*, the speed of an expanding OF was varied sinusoidally (0.017 Hz, 1 sine duration = 60 s), from 0 to 2 times the individual's comfortable walking speed, for a total duration of 5 minutes. The sine wave was preceded and ended by 30 seconds of OF matching comfortable speed. In *Experiment 2*, subjects were instructed to walk at a comfortable pace (control trials) on the self-paced treadmill through a 13 m-long virtual corridor comprising of 2 doorways located 10 m apart. Subjects started walking 1.5 m before the first door and ended at 1.5 m after the second door when a stop sign appeared. Every control trial was followed by a test speed trial, during which expanding OFs corresponding to 0.25, 0.5, 0.75, 1.00, 1.25, 1.50, 1.75 and 2 times the individual's comfortable speed were randomly presented. For each test speed trial, subjects were instructed to cover the corridor distance within the same time span as the previous control trial.

Distance travelled and walking speed were provided by the treadmill tachometer and recorded at 100 Hz using the Caren-2^® ^system (Motek) that also manipulated the OF speeds. In addition, the treadmill speed signal was recorded at 360 Hz using the Vicon-512 A-D system. Speed signals were low-pass filtered at 0.02 Hz (second-order Butterworth, dual pass for zero lag).

### Data Analysis

For Experiment 1, the strength of the relationship and phase lag between the OF speed and walking speed signals were analyzed by means of cross-correlations. For Experiment 2, the mean walking speed over the 10 m separating the 2 doorways was calculated for every trial. Changes in walking speed for the speed trials were expressed as a ratio of the mean walking speed in the test trial over the control trial (gait speed ratio). The slopes between gait and OF speed ratios were obtained using linear regressions. All outcome variables, including cross-correlation coefficients, phase lags and slopes were compared between subjects with stroke and healthy controls using Student t-tests for independent samples. The relationship of these variables with the subjects' comfortable gait speeds was also quantified using Pearson correlation coefficients. Statistical analyses were carried out in Statistica 7.0 ^® ^and the level of significance was set p < 0.05.

## Results

### Experiment 1

**Figure 2:**
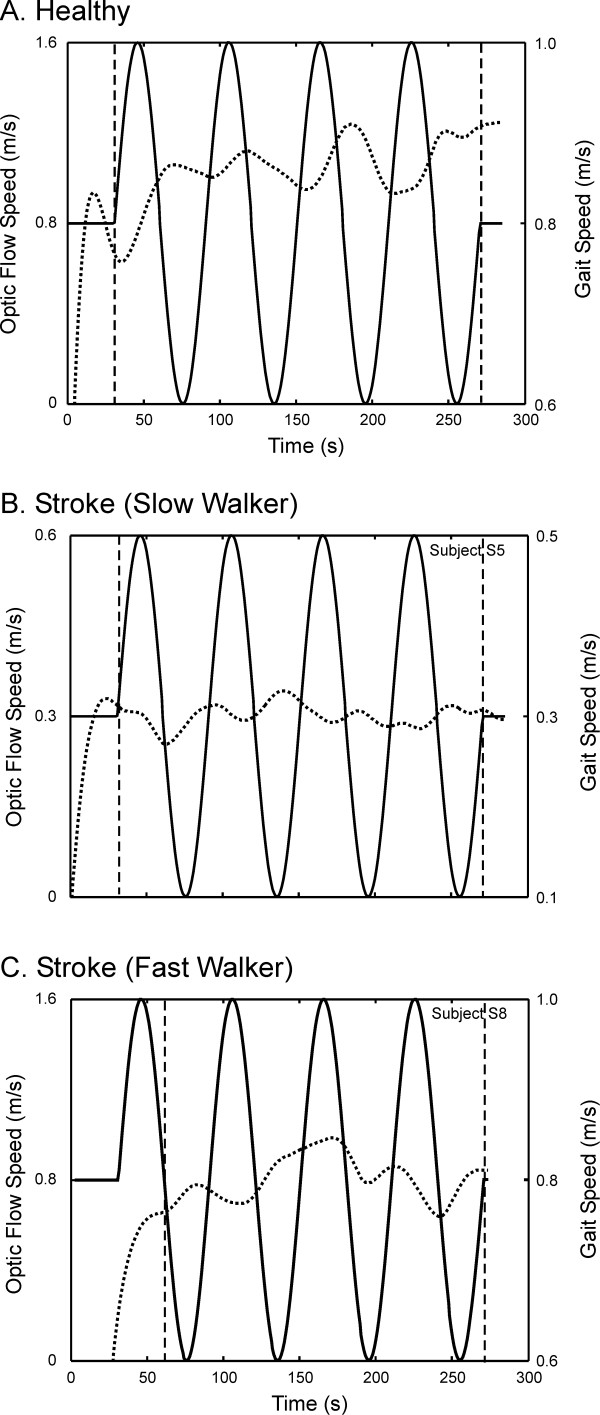
Modulation of gait speed (dotted lines) as a function of optic flow speed (plain lines) in a healthy subject (A) and subjects with stroke presenting with a slow (B) and a faster walking speed (C). Data were analyzed once the subjects reached their comfortable speed, as indicated by the vertical dashed lines. Left and right y-axes are for optic flow speed and gait speed, respectively. Note the different y-axis scales amongst the subjects.

**Figure 3:**
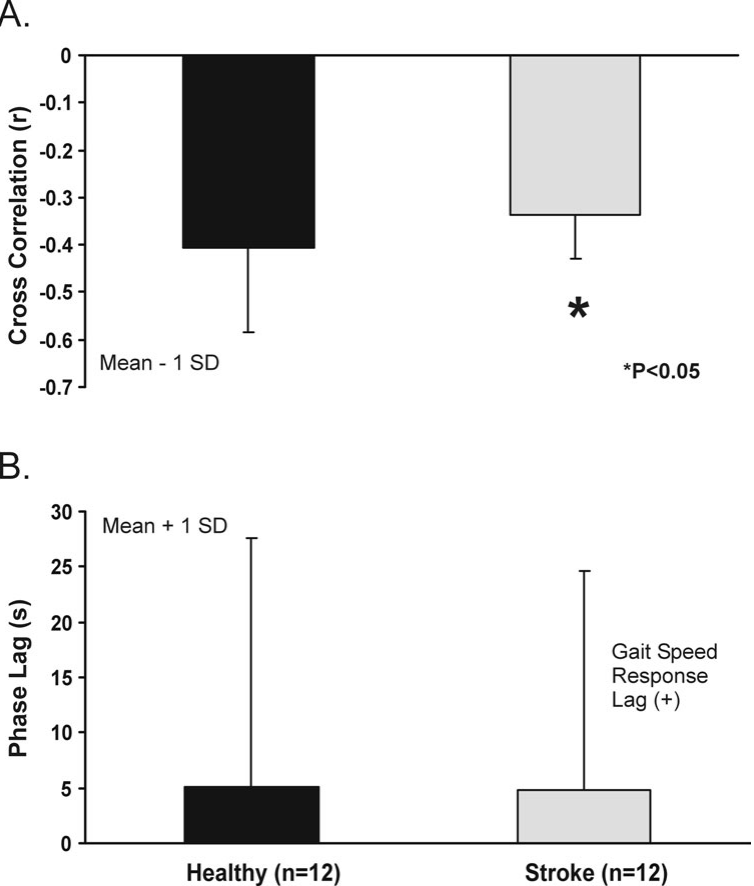
Mean cross-correlation coefficients (A) and phase lags (B) calculated between gait speed and optic flow speed in the healthy subjects and subjects with stroke.

Examples of modulation of walking speed in response to an OF of continually changing speed are illustrated for a healthy subject and subjects with stroke in Figure 2. In healthy subjects, gait speed was modulated out-of-phase with respect to OF speed, such that faster walking speeds were observed at slower OFs, and slower walking speeds at faster OFs. Subjects with stroke presented either with less modulation or changes in walking speed (Figure 2B), or patterns of modulation that varied from out-of-phase to in-phase (Figure 2C). On average, cross-correlation analyses revealed negative correlation coefficients between gait speed and OF speed for both groups (Figure 3). The strength of this relationship, however, was weaker (p < 0.05) in subjects with stroke than in healthy controls. Similar phase lags were observed in both groups, with a speed response lagging behind the changes in OF speed by 5.1 s and 4.8 s, respectively, in the healthy and stroke groups. Neither the cross-correlation coefficients (R^2 ^= 0.08, P = 0.4) nor the phase lags (R^2 ^= 0.08, P = 0.4) were associated with the comfortable overground walking speed post stroke.

### Experiment 2

**Figure 4:**
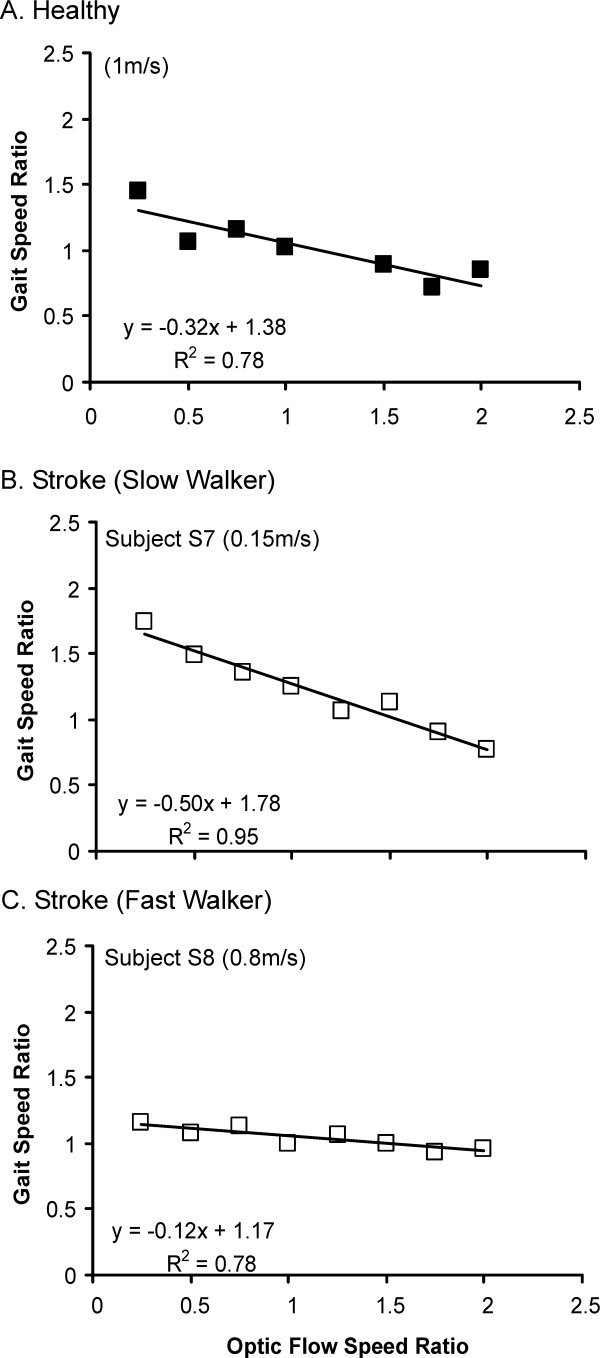
Changes in gait speed as a function of changes in optic flow speed in a healthy subject (A) and subjects with stroke presenting with a slow (B) and fast (C) walking speed. Both gait speed and optic flow speed were expressed as ratios of the actual speed observed in the test trial as compared to control trial with matching gait and OF speeds.

**Figure 5:**
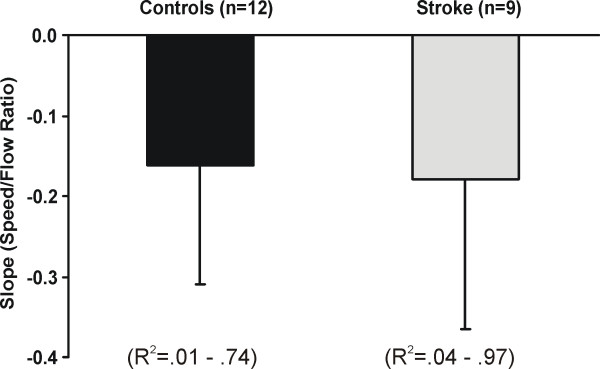
Mean slopes calculated between gait speed and optic flow speed ratios in healthy subjects and subjects with stroke. The ranges of the coefficients of determination (R^2^) are indicated for each group.

Figure 4 represents examples of changes in walking speed as a function of changes in OF speed, as measured during Experiment 2. In healthy subjects and subjects with stroke, a negative linear relationship was generally observed between gait speed and OF speed, such that subjects walked faster at slower OFs. The gain of this relationship, as quantified by the slope between gait speed ratio vs. OF speed ratio, was similar between subjects with stroke and healthy controls (p > 0.05) (Figure 5). On average, the highest increments in speed observed at slow OFs reached 1.32 ± 0.25 and 1.44 ± 0.38 times comfortable gait speed values, respectively, in the healthy and stroke groups. Subjects with stroke who walked at slower comfortable speeds displayed steeper modulation slopes (R^2 ^= 0.53, p < 0.05) and higher ratios of speed increment (R^2 ^= 0.78, p < 0.05) than those who walked at faster initial speeds.

## Discussion and Conclusions

The purpose of this study was to investigate the ability of persons with stroke to modulate their speed of walking in response to changing OF speeds. The main findings are that subjects with stroke present with alterations in their speed modulation response to changing OF speeds. However, when instructed to use OF speed information to scale their walking speed, they display patterns of modulation that are similar to those observed in age-matched healthy subjects.

Results of the present study demonstrate that walking speed is modulated out-of-phase with OF speed in healthy subjects, which is consistent with previous studies [6-8], despite the fact that expanding OFs were simulated by a meaningful and rich virtual environment in the present study. Previous research used gratings composed of dots, spots or diamonds oscillating to form contracting and expanding OFs [6-8]. The observed non-volitional changes in gait speed induced by changes in OF speed in our healthy age-matched controls, although consistent, were of small magnitude. According to Varraine and collaborators [8], this consistency combined with the fact that OF speed variations are not fully compensated by changes in gait speed, would argue in favor of a 'low level phenomenon' that is not under voluntary control. Present findings also revealed that gait speed responses were lagging behind the changes in OF speed. Such a lag may be explained by the latency of OF perception added to the latency for the mechanical transformation of the segmental kinematics.

Results from Experiment 2, in which subjects voluntarily attempted to walk a fixed distance within the same time span while exposed to OFs of different speeds, yielded larger walking speed changes than when exposed to the sinusoidally changing OF speeds in Experiment 1. This difference was observed despite the fact that the range of OF speed was smaller in Experiment 2 (0.25 to 2 times the individual's comfortable speed) than during Experiment 1 (0 to 2 times). The larger modulation observed in Experiment 2 may be attributed to the fact that information from constant OF speeds is easier to perceive and integrate than continually changing speeds, while a cognitive intentional process was also involved. The fact that the walking distance was shorter and that perception of time (duration required to walk the hallway) may have been a useful source of information to complete the task may also have contributed to the larger gait speed modulation responses observed in the second experiment. Gait speed adaptation did not fully compensate the mismatch with OF speed, even by our healthy aged-matched subjects, as indicated by slope values that did not approach -1.0. This partial compensation suggests the involvement of other sensorimotor transformations based on an internal representation and the central nervous system's integration of other sensory cues, such as leg proprioceptive information.

Present results also showed that subjects with stroke displayed abnormalities in their non-volitional or unintentional modulation of walking speed. Both animals [20-22] and human studies [23-26] suggest that optic flow perception involves not only the occipital, but also parietal and temporal cortical areas. Overall, as the complexity of the stimulus increases (first vs. second order stimuli), increasing neural networks involving more anterior brain regions are recruited [27-29]. Persons with a stroke in the temporal lobe or in adjacent regions of the frontal or parietal lobes manifest abnormally high thresholds for the discrimination of global motion direction [17]. Whether they also present with higher discrimination thresholds for optic flow speed is still unclear. We have also shown that subjects with stroke present with a difficulty in integrating multiple sensory information, such as those induced during a rapid voluntary head turn while standing or walking [30,31]. The altered modulation response observed in the subjects with stroke could thus be explained by an altered discrimination of OF speed and/or a reduced ability to integrate multiple sources of sensory information.

It is also noteworthy that the down-regulation response observed in the subjects with stroke differs from the up-regulation response observed in subjects with Parkinson's disease, which manifests as an abnormally large modulation in walking speed in response to changing OF speed [10]. This finding in Parkinson's disease was interpreted as a higher reliance on visual kinesthesia, possibly due to an altered proprioceptive guidance of movement (proprioceptive kinesthesia). In the present study, none of our subjects with stroke showed abnormally large responses, which could suggest that they do not rely on visual kinesthesia as much as in Parkinson's disease. It is also possible that the characteristics of the OF substantially influences the speed modulation response. Hence, the use of an expanding OF in the present study may not be comparable to an OF that consists of both expansions and contractions in the study with Parkinson Disease subjects [10]. In the early exploratory phase of this study, we had tested an OF that oscillated between expansion and contraction, simulating forward and backward self-motion. That type of stimulus caused a few subjects with stroke to behave differently in an attempt to walk backwards or completely stop walking with the contracting OFs (unpublished observations). For feasibility and safety reasons, as well as for the purpose of generalization of results to real life situations, it was decided in the present study that only expanding OFs simulating forward translation would be tested.

Results from Experiment 2 also reveal that subjects with stroke, once cognitively though intentionally involved in the task, produce speed modulation responses that are similar to that observed in the age-matched healthy subjects. The fact that the stroke group performed within the norms in the second experiment, but not during the first experiment, suggests that the two tasks involved different stimuli (changing vs. constant speed, reliance on time perception) and that incorporating a volitional component yields more accurate, yet not fully adapted, speed modulation responses. Most importantly, the adaptability of subjects with stroke in the second experiment provide a strong rationale to incorporate the manipulation of OF speed in a virtual-reality training paradigm that aims at promoting faster walking speeds. Present results indicate that subjects with stroke could instantaneously increase their walking speed by 1.44 times, which represents an increase close to 50%. To our knowledge, it is the first evidence that a virtual environment simulating OF could induce such large changes in gait speed in subjects with stroke. Before such an approach can be implemented in a virtual reality-based gait rehabilitation program, further work is needed to determine whether habituation occurs in the intentional speed modulation paradigm, as for the unintentional paradigm [7], and the carry-over effects to overground locomotion.

## Competing interests

The author(s) declare that they have no competing interests.

## Authors' contributions

Lamontagne conceived and carried out the experiment and analyses presented in this paper. She also drafted the manuscript. Fung and McFadyen contributed to the design of the study, data analyses and reduction, as well as revision of the manuscript. Faubert contributed to the design and psychophysical aspects related to optic flow perception. All authors read and approved the final manuscript.
